# Supplementary material for: Extrinsic and intrinsic signals converge on the Runx1/CBFβ transcription factor for nonpeptidergic nociceptor maturation
Source: eLife. 2015 Sep 29;4:e10874. doi: 10.7554/eLife.10874 (PMC4657622; doi:10.7554/eLife.10874)
Supplement: Supplementary file 1. — DOI:http://dx.doi.org/10.7554/eLife.10874.019 [file elife-10874-supp1.pdf]

Table S1. Microarray analysis of genes that are differentially expressed in E16.5 DRGs of control and *Runx1 CKO* animals. Shown are genes with a fold change greater than or equal to 1.5, and a p-value less than or equal to 0.05. Genes that exhibited reduced or increased expression in *Runx1 CKO* DRGs relative to control are shown in table S1.1 and S1.2, respectively.

Table S1.1

Probe Set ID	Gene Symbol	Gene Title	Fold Change	P-value
1443392_at	Trpv1	transient receptor potential cation channel, subfamily V, member 1	3.855363	0.000364
1418723_at	Lpar3	lysophosphatidic acid receptor 3	3.150196	0.017528
1424633_at	Camk1g	calcium/calmodulin-dependent protein kinase I gamma	2.943805	0.003527
1448459_at	Kcnip1	Kv channel-interacting protein 1	2.265259	0.03317
1443959_at	Tmem72	transmembrane protein 72	2.163699	0.003249
1438112_at	9430021M05Rik	RIKEN cDNA 9430021M05 gene	2.14351	0.007824
1436100_at	Sh2d5	SH2 domain containing 5	2.11848	0.03425
1441363_at	Frmpd3	FERM and PDZ domain containing 3	2.078189	0.039236

1435772_at	Kif21b	kinesin family member 21B	2.015971	0.000204
1416456_a_at	Chia	chitinase, acidic	2.005046	0.028087
1416785_at	Kcnip1	Kv channel-interacting protein 1	1.996721	0.002743
1419654_at	Tle3	transducin-like enhancer of split 3, homolog of Drosophila E(spl)	1.934638	0.049115
1428074_at	Tmem158	transmembrane protein 158	1.902171	0.041199
1420564_at	Insrr	insulin receptor-related receptor	1.868479	0.009468
1452263_at	Slc35f4	solute carrier family 35, member F4	1.823904	0.016667
1451062_a_at	Pex5l	peroxisomal biogenesis factor 5-like	1.819641	0.029577
1434635_at	Rph3a	rabphilin 3A	1.803495	0.036091
1455000_at	Gpr68	G protein-coupled receptor 68	1.779183	0.033036
1450224_at	Col4a3	collagen, type IV, alpha 3	1.762817	0.021838
1438160_x_a	Slco4a1	solute carrier organic anion	1.745016	0.04389

t		transporter family, member 4a1		8
1425483_at	LOC100044677 /// Tox	similar to thymus high mobility group box protein TOX /// thymocyte selection-as	1.744519	0.026119
1419655_at	Tle3	transducin-like enhancer of split 3, homolog of Drosophila E(spl)	1.716283	0.026226
1429805_at	Myo1a	myosin IA	1.714405	0.000569
1433988_s_at	C230098O21Rik	RIKEN cDNA C230098O21 gene	1.707104	0.044154
1440056_at	---	---	1.672219	0.001931
1424923_at	Serpina3g	serine (or cysteine) peptidase inhibitor, clade A, member 3G	1.65806	0.002164
1421037_at	Npas2	neuronal PAS domain protein 2	1.654117	0.027774
1445941_at	---	---	1.650902	0.02853
1417542_at	Rps6ka2	ribosomal protein S6 kinase, polypeptide 2	1.646436	0.020774
1422710_a_at	Cacna1h	calcium channel, voltage-dependent, T type, alpha 1H subunit	1.629623	0.024766
1436013_at	Gsg1l	GSG1-like	1.602431	0.002997

1417392_a_at	Slc7a7	solute carrier family 7 (cationic amino acid transporter, y+ system), member 7	1.598559	0.048681
1453801_at	Them5	thioesterase superfamily member 5	1.595386	0.021385
1431852_at	A730035I17Rik	RIKEN cDNA A730035I17 gene	1.584555	0.000854
1441298_at	---	---	1.565934	0.039893
1450174_at	Ptprt	protein tyrosine phosphatase, receptor type, T	1.557751	0.027241
1430159_at	5830408C22Rik	RIKEN cDNA 5830408C22 gene	1.520074	0.039318
1456047_at	LOC433466 /// Pla2g4b	phospholipase A2, group IVB (cytosolic)	1.517543	0.049284
1438055_at	Rarres1	retinoic acid receptor responder (tazarotene induced) 1	1.511225	0.02733
1457128_at	AL024213	expressed sequence AL024213	1.508931	0.040555

Table S1.2

**Probe Set ID**	**Gene Symbol**	**Gene Title**	**Fold Change**	**P -Value**

1455931_at	Chrna3	cholinergic receptor, nicotinic, alpha polypeptide 3	4.589469	0.030475
1417256_at	Mmp13	matrix metallopeptidase 13	3.542615	0.019814
1433607_at	Cbln4	cerebellin 4 precursor protein	3.482604	0.011625
1435424_x_at	---	---	3.416324	0.001092
1433551_at	Vat1l	vesicle amine transport protein 1 homolog-like (T. californica)	3.278425	0.018548
1440484_at	Unc5d	unc-5 homolog D (C. elegans)	3.169972	0.002508
1441329_at	Galr1	galanin receptor 1	3.067817	0.000843
1452010_at	Chrna3	cholinergic receptor, nicotinic, alpha polypeptide 3	2.952662	0.000112
1451263_a_at	Fabp4	fatty acid binding protein 4, adipocyte	2.837796	0.035
1417023_a_at	Fabp4	fatty acid binding protein 4, adipocyte	2.716484	0.034395
1439272_at	Lcorl /// LOC100046011	ligand dependent nuclear receptor corepressor-like /// hypothetical protein LOC1	2.610205	0.04198

1416468_at	Aldh1a1	aldehyde dehydrogenase family 1, subfamily A1	2.508498	0.046711
1437695_at	Prokr2	prokineticin receptor 2	2.473296	0.045341
1443365_at	Htr4	5 hydroxytryptamine (serotonin) receptor 4	2.377891	0.043301
1430107_at	Acbd7	acyl-Coenzyme A binding domain containing 7	2.213863	0.006569
1417680_at	Kcna5	potassium voltage-gated channel, shaker-related subfamily, member 5	2.065996	0.033749
1439887_at	---	---	2.048765	0.007273
1449254_at	Spp1	secreted phosphoprotein 1	2.038316	0.044844
1418304_at	Pcdh21	protocadherin 21	2.020208	0.011017
1457008_at	Chrnb4	cholinergic receptor, nicotinic, beta polypeptide 4	1.957697	0.016487
1419756_at	Dgkg	diacylglycerol kinase, gamma	1.914247	0.039183
1436761_s_at	1200015N20Rik	RIKEN cDNA 1200015N20 gene	1.891189	0.01325
1456233_at	---	---	1.871748	0.026841

1440531_at	Rbm11	RNA binding motif protein 11	1.870702	0.045852
1456609_at	Camk2n1	calcium/calmodulin-dependent protein kinase II inhibitor 1	1.868836	0.002939
1443322_at	---	---	1.868507	0.018301
1437883_s_at	---	---	1.84781	0.047311
1452004_at	Calca	calcitonin/calcitonin-related polypeptide, alpha	1.846593	0.034994
1442379_at	EG574403	predicted gene, EG574403	1.834135	0.049862
1436444_at	6030405A18Rik	RIKEN cDNA 6030405A18 gene	1.819695	0.01243
1436493_at	BB181834	expressed sequence BB181834	1.814729	0.001058
1437262_x_at	Bcas2	breast carcinoma amplified sequence 2	1.813717	0.043032
1424679_at	Mab21l1	mab-21-like 1 (C. elegans)	1.8063	0.012424
1438698_at	Tmem132c	transmembrane protein 132C	1.80257	0.012791
1426222_s_at	Vwa5a	von Willebrand factor A domain containing 5A	1.799035	0.008401

1434150_a_at	Higd1c /// Mettl7a1 /// Mettl7a2	HIG1 domain family, member 1C /// methyltransferase like 7A1 /// methyltransferase	1.793921	0.0031
1456397_at	Cdh4	cadherin 4	1.789226	0.006951
1437781_at	Insm2	insulinoma-associated 2	1.785904	0.043102
1445247_at	C530044C16Rik	RIKEN cDNA C530044C16 gene	1.77605	0.008473
1460244_at	Upb1	ureidopropionase, beta	1.764131	0.005432
1451033_a_at	Trpc4	transient receptor potential cation channel, subfamily C, member 4	1.75628	0.038596
1449251_at	Ndp	Norrie disease (pseudoglioma) (human)	1.749063	0.020795
1460033_at	C030002C11Rik	RIKEN cDNA C030002C11 gene	1.73966	0.035207
1437800_at	Edaradd	EDAR (ectodysplasin-A receptor)-associated death domain	1.738899	0.008914
1423571_at	S1pr1	sphingosine-1-phosphate receptor 1	1.718975	0.040307
1423016_a_at	Gypa	glycophorin A	1.709037	0.048106

1429123_at	Rab27a	RAB27A, member RAS oncogene family	1.70457	0.022827
1434297_at	E130304F04Rik /// LOC100040601	RIKEN cDNA E130304F04 gene /// hypothetical protein LOC100040601	1.69251	0.003604
1448421_s_at	Aspn	asporin	1.656201	0.028697
1419599_s_at	Ms4a6d	membrane-spanning 4-domains, subfamily A, member 6D	1.654013	0.043855
1445838_at	---	---	1.646932	0.046888
1449033_at	Tnfrsf11b	tumor necrosis factor receptor superfamily, member 11b (osteoprotegerin)	1.644202	0.034106
1421854_at	Fgl2	fibrinogen-like protein 2	1.640142	0.000127
1427313_at	Ptgir	prostaglandin I receptor (IP)	1.636612	0.018072
1437989_at	Pde8b	phosphodiesterase 8B	1.624623	2.04E-05
1426937_at	6330406I15Rik	RIKEN cDNA 6330406I15 gene	1.601092	0.049248
1418454_at	Mfap5	microfibrillar associated protein 5	1.593589	0.021051

1442082_at	C3ar1	complement component 3a receptor 1	1.57566	0.042136
1418511_at	Dpt	dermatopontin	1.573438	0.026282
1429197_s_at	Rabgap1l	RAB GTPase activating protein 1-like	1.557097	0.020342
1419468_at	Clec14a	C-type lectin domain family 14, member a	1.548326	0.012029
1417012_at	Sdc2	syndecan 2	1.538801	0.032822
1435616_at	Cyp20a1	cytochrome P450, family 20, subfamily A, polypeptide 1	1.534345	0.048825
1457137_at	---	---	1.531957	0.04647
1424229_at	Dyrk3	dual-specificity tyrosine-(Y)-phosphorylation regulated kinase 3	1.524185	0.037066
1426514_at	4631426J05Rik	RIKEN cDNA 4631426J05 gene	1.50781	0.047725
1427319_at	A230046K03Rik	RIKEN cDNA A230046K03 gene	1.506691	0.036402
1445767_at	Ptprd	protein tyrosine phosphatase, receptor type, D	1.501627	0.001025
